# Selenium Toxicity from a Misformulated Dietary Supplement, Adverse Health Effects, and the Temporal Response in the Nail Biologic Monitor

**DOI:** 10.3390/nu5041024

**Published:** 2013-03-28

**Authors:** John Steven Morris, Stacy B. Crane

**Affiliations:** 1 Research Reactor Center, University of Missouri, Columbia, MO 65211, USA; E-Mail: cranes@missouri.edu; 2 Division of Research Services, Truman Memorial Veterans Hospital, Columbia, MO 65211, USA

**Keywords:** selenium supplementation, toxicity, selenosis symptoms, fingernails, toenails, human study

## Abstract

Use of dietary supplements in the U.S. has increased steadily over the last 25 years. While misformulation is uncommon, the consequences can be serious. A March 2008 voluntary market recall removed supplement products responsible for the most serious selenium toxicity outbreak that has occurred in the U.S. We quantified selenium concentrations in the misformulated supplement products, measured the temporal response in the nail biologic monitor, and associated exposure to self-reported selenosis symptoms. Subjects recruited through state health departments and referrals provided samples of the misformulated supplement products, exposure information, monthly toenail and or fingernail clippings or onycholysitic nail fragments, and listed their newly onset adverse health effects attributed to selenium toxicity. Ninety-seven subjects enrolled and submitted at least one test sample. Peak selenium concentrations (up to 18.3 and 44.1 μg/g for toenails and fingernails, respectively) were measured. Multiple samples (52 total) of all six recalled supplement lots were analyzed ranging from 22,300 to 32,200 μg selenium per daily dose. Average consumption was 30.9 ± 13.9 doses; 73 subjects provided follow-up data on selenosis symptoms at 2.50 ± 0.14 years. Nail samples accurately reflect exposure in this selenium toxicity outbreak, which resulted in long-term/permanent adverse health effects.

## 1. Introduction

Selenium (Se) is a required trace-element micronutrient having a recommended dietary allowance (RDA) of 55 to 70 micrograms (μg) per day [1]. The determination of the RDA has typically been based on the Se-intake needed to saturate glutathione peroxidase activity. More recently, saturation of the serum selenoprotein P concentration, which occurs at a higher Se intake, has been proposed as a better estimate of the Se requirement [2]. There are at least 25 mammalian selenoproteins [3] having biological functions or expressions known to various degrees [4,5]. The discovery of the essentiality of Se in 1957 [6], the recognition of selenocysteine (Sec) as the 21st amino acid and the mechanism by which Sec is inserted in mammalian proteins [7,8], and the implication of selenoproteins in the etiologies of cancer, and cardiovascular disease [9,10,11], have generated substantial interest in the scientific community that has been translated through the popular press to the general public, often promoting an increased Se intake through supplementation. Se is also known to be toxic with a narrow range separating chronic conditions of deficiency and toxicity. Studies of higher than normal Se intakes in a seleniferous area in the U.S. [12] and Enshi County of Hubei Province of China [13] led to the establishment of the Tolerable Upper Limit (UL), No Observed Adverse Effect Level (NOAEL) and the Lowest Observed Adverse Effect Level (LOAEL) for Se at 400, 800 and 913 μg/day, respectively [1]. These prudently conservative limits are supported by a study in which 24 men with prostate cancer were treated with selenized yeast at either 1600 or 3200 μg/day for 12 months (most subjects). Modest selenosis symptoms (brittle hair and nails, stomach upset, dizziness) were observed primarily, but not exclusively, in the high-dose group [14].

The reported use of dietary supplements (at least once in the past month), based on the National Health and Nutrition Examination Survey (NHANES), has increased from 28% and 38% in 1971–1974 to 44% and 53% in 2003–2006 for males and females, respectively [15]. Males and Females combined in the 19–30 years, 31–50 years, 51–70 years and ≥71 years age groups had a steady increase in the use of Se-containing supplements of 16%, 21%, 30% and 32%, respectively, in the 2003–2006 survey period [15]. In a 1999 study biased toward 3575 health-conscious adults living in 111 of Missouri’s 114 counties, we found Se supplement use to be over represented at 42.7% and 39.1% with average doses of 81.4 μg/day and 73.4 μg/day for males (56.4 ± 14.2 years; *n* = 1635) and females (53.7 ± 14.1 years; *n* = 1940), respectively [16].

An analysis of 15 popular Se supplement products, including both organic and inorganic chemical forms, purchased at two time points (June 2000 and January 2003) found mean (*n* ≥ 10 doses) Se concentrations higher than the label value for most products tested ranging from differences of −7.2% to +39.2% in 2000 and −13.4% to +19.5% in 2003. Individual doses ranged from 83% to 250%, in 2000, and 78% to 160%, in 2003, of the label value [17], which does not inspire confidence in the manufacturing practices employed by some of the supplement companies. The highest Se content (322 μg) measured in any single individual dose did not exceed the UL of 400 μg/day. However, the UL likely would have been exceeded when dietary Se is included. 

While uncommon, several episodes of acute Se toxicity resulting from misformulated supplement products have been previously reported. In 1983–1984, a misformulated Se supplement, containing 27,300 μg Se per tablet, was taken by 13 individuals resulting in selenosis symptoms including nausea, abdominal pain, diarrhea, peripheral neuropathy, fatigue, irritability and hair and nail changes. One female subject who consumed 77 pills over a 2.5 months period had a nail Se concentration of 8.1 μg/g, approximately 10 times normal [18,19]. In 1996, a 36 years old man consumed misformulated Se tablets (content not specified) and experienced diarrhea, fatigue, tingling in his extremities and hair loss [20]. 

The practice of taking Se supplements with no knowledge of baseline Se status is both common and ill advised. The daily Se intake for adults in the U.S. has been measured at 81 ± 41 μg/day [21] and 79–104 μg/day [22] from diet analyses and estimated at 60–220 μg/day from dietary recall and food composition tables [15,23,24,25]. For many, adding a Se supplement to a U.S. diet already adequate in Se, along with the modest to elevated over-potency of some of these products, increases Se intake to the UL of 400 μg/day or greater and may be a contributing factor to the high number of consumer complaints, estimated at 0.4%, based on reports from users of multivitamin-multimineral supplements in the USFDA Health and Diet Survey [26].

A daily Se intake at the UL, while not causing observed selenosis symptoms, may nevertheless increase the long-term risk of chronic disease for at least some of the population. The U-shaped curve, with its deficient and toxic arms separated by a safe intake range, was first proposed for nutrient intake in 1912 by Bertrand [27] and then largely ignored until reintroduced by Mertz with an emphasis on trace-element nutrients [28,29]. More recently, the U-Curve has been discussed for micronutrients [30] and specifically for multivitamin-multimineral supplements [31] relevant to the potential for harm resulting from higher intakes previously considered benign. For example, Se and prostate cancer has been reviewed in a meta-analysis of 12 studies [32] in which the authors suggest the mixed findings may follow a U-shaped curve as suggested by others for prostate and other cancers [9,10,33], and in a canine prostate cancer model in which apoptosis and DNA damage were associated with Se status through a U-shaped response to Se supplementation [34]. A similar trend indicating that the upper limit of healthy Se intake is well lower than the UL is emerging for peripheral arterial disease [35], hypertension [36], serum lipids [37], cardiovascular disease [11,38,39,40], and diabetes [41,42]. The Se requirement, whether it is uniformly met through dietary Se intake alone in the U.S., or if there exists some fraction of the population who would benefit from Se supplementation, is a complex issue involving chemical form, absorption, gene expression, variability in Sec insertion efficacy and selenoprotein polymorphisms [4,5].

Against this backdrop, the U.S. Food and Drug Administration (FDA) issued a 27 March 2008, news release announcing a distributor recall of “Total Body Formula” (TBF) and “Total Body Mega Formula” supplement products and warning consumers that these products had caused serious adverse reactions including “significant hair loss, muscle cramps, diarrhea, joint pain and fatigue” within 10 days of their consumption [43]. In a second news release on 9 April 2008, the FDA reported that samples of the TBF supplement products “contain extremely high levels of Se—up to 40,800 μg per recommended serving” instead of the 200 μg intended [44]. On 14 April 2008, the Centers for Disease Control and Prevention (CDC) issued a CDC Health Advisory that described the individual TBF supplement products; provided the 6 misformulated lot numbers; alerted consumers of the adverse health effects; and identified product distribution to include Alabama, California, Florida, Georgia, Kentucky, Louisiana, Michigan, Missouri, New Jersey, North Carolina, Ohio, Pennsylvania, South Carolina, Tennessee, Texas, Virginia, and also via the Internet [45]. In a final 1 May 2008, news release, the FDA announced that in addition to the extremely high levels of Se that had caused 201 confirmed selenosis cases, the TBF supplement products also contained 3426 μg of chromium per recommended serving, 17 times the RDA [46].

The Total Body Formula products were distributed by Total Body Essential Nutrition, Inc., Woodstock, Georgia. To our knowledge, this company is no longer in business and their products are no longer available. Approximately 1200 bottles of the misformulated TBF products were manufactured in September 2007 [47] in six lots. At the time of the Se toxicity outbreak, largely in January through March of 2008, the TBF supplements were described in product literature as “a complete full-spectrum dietary supplement for the entire family in liquid form” containing “16 essential vitamins, 70 colloidal minerals, 18 amino acids, 3 essential fatty acids, whole grape extract, and CoQ10”. The so-called “Mega Formula” product in addition contained “whole food complex, aloe, and bee pollen”. Product labels, in addition to the colloidal minerals, also included 12 supplemented minerals and trace elements along with their daily values including Se at 200 μg per 1-ounce dose and a daily value of 290%. The products are water based and were manufactured in several citrus flavors and were distributed primarily in the Southeastern U.S. through small-market nutritional supplement stores, via the Internet and from at least one medical clinic. Total Body Essential Nutrition contracted with an outside company to blend the ingredients, bottle, and label the TBF products. A third company produced the vitamin-mineral dry mixture including Se as sodium selenate (There is disagreement in product-related documents regarding the chemical form of selenium in the TBF products. On at least some of the product labels selenium is listed as sodium selenite. The documents, including the certificate of analysis provided by the company that produced the vitamin-mineral dry mixture, list the selenium as sodium selenate. We have assumed that the chemical form listed on the dry-mixture product documents, sodium selenate, to be correct.). According to multiple documents, provided by the company that manufactured the dry mixture, including the initial certificate of analysis, the “Label Claim” for sodium selenate (presumably that quantity per individual dose) in all the documents was given as 112.5 mg. Manufactured at this formulation would result in a Se concentration of 47,000 μg per dose, 235 times the intended dose and acutely toxic. In March 2008, in response to emerging health complaints from TBF product users, the quality director at the company that had produced the dry mixture, without undertaking an analysis of the dry mixture, changed the label claim mass units for sodium selenate on the certificate of analysis from milligrams to micrograms erroneously asserting that the original specification of milligrams was a typographical error. Throughout the production of the misformulated TBF supplement products, there were opportunities for each of the three companies involved to avoid the Se toxicity outbreak had adequate quality control procedures been in place and followed at any one of the three companies.

These misformulated TBF supplement products, subject to the voluntary recall, were responsible for the most serious Se toxicity outbreak that has occurred in the U.S. The objectives of the study reported here are: (1) To quantify the Se content in multiple samples from all 6 misformulated lots of the liquefied TBF supplement products and determine the Se distribution among the dissolved and suspended phases. (2) To enroll subjects who had consumed the misformulated TBF supplement products and measure the temporal Se response in fingernail (FN) and/or toenail (TN) specimens collected on a monthly basis, or in onycholysitic nail fragments that had spontaneously detached. Of particular interest is the dose response relative to both the peak Se concentration reached in the nail biologic monitor and also the Se concentration measured in the restored baseline. (3) To collect self-reported symptoms, their persistence, and impact on day-to-day life.

## 2. Experimental Section

### 2.1. Subjects and Nail Samples

The misformulated TBF products were manufactured in September 2007 and commercially distributed to the general public beginning in January 2008 until their voluntary recall on 27 March 2008. To measure the temporal response in the nail biologic monitor to the acute Se exposure in subjects already exposed, an expedited review was requested and the project, titled Selenium Supplement Intoxication Study (SSIS), was approved by the University of Missouri Health Sciences Institutional Review Board on 8 June 2008. Subjects were then recruited through state health departments and referrals. A total of 97 subjects enrolled in the SSIS and provided at least 1 biomonitor sample. The initial group of 43 subjects had previously contacted a state health department in response to the FDA News Releases [43,44,46], the CDC Health Advisory [45], or information obtained from other individuals aware of the Se toxicity outbreak. These 43 subjects gave permission for their names and contact information to be provided to the principal investigator of the SSIS and they were subsequently invited to join the study. Each potential participant was mailed a letter describing the SSIS, an enrollment form and questionnaire, a consent form, a lay description of the use of the TN biologic monitor to measure Se intake and status, instructions for TN sample collection, 12 pre-labeled sample envelopes, and 12 pre-addressed and postage paid (USPS) return envelopes. The introductory letter explained the objectives as described previously and requested a set of TN clippings from as many TNs as feasible to be collected beginning immediately and subsequently on an approximate monthly schedule for an expected 12 months study period. We explained that the TN samples provided would be analyzed for Se and the results would be provided by mail on a timely basis at no cost to the participants.

Of these initial 43 individuals expressing interest, 36 completed the enrollment questionnaire and consent form and joined the SSIS. Of these 36 subjects, 29 were able to either provide a TN clipping sample on an approximate monthly basis or had retained one or more onycholysitic nail fragments that had spontaneously detached and could be segmented to represent the equivalent of 3 or more monthly clipping samples. In this group, the mean ± s.d. lapsed time from the last use of a misformulated TBF product to first TN sample collection was 129 ± 68 days (median = 115 days). In all 29 subjects this first sample collection occurred prior to the eventual peak Se concentration. Consequently, we were able to measure the temporal response in the nail monitor including both the appearance of the peak Se concentration and its return to a post-exposure baseline for these 29 subjects. The remaining 7 subjects had lost their nails prior to joining the SSIS but had not retained them. These subjects ultimately provided new-growth nails whenever possible; however it was not possible to definitively establish the peak toenail selenium (TNSe) concentration in these 7 subjects.

Throughout the remainder of 2008 and 2009, 61 additional subjects, who were referred to the SSIS by word-of-mouth and through 4 law offices representing one or more individuals who had consumed the misformulated TBF supplement products, joined the study. Within this subset, the mean ± s.d. lapsed days from the last use of a misformulated TBF product to the first TN sample collection was 457 ± 53 days (median = 466 days). For these subjects the peak TNSe concentration had already occurred. Where validated, these long-term post-exposure samples were included in the determination of the post-exposure baseline Se status.

Two of the male subjects, who lost all 10 FNs, which had occurred prior to their learning of the SSIS, nevertheless had the presence of mind to save these fragments identified by digit and detachment date. These fragments were segmented and analyzed for Se to provide individual chronologies for each of the 10 FNs. One of these subjects has continued to provide new-growth clippings from each FN and TN at least once a month. These have been analyzed for Se to extend the temporal response for this subject to 1602 days (4.386 years) post exposure.

In total, 97 subjects joined the SSIS and are described in Table 1.

**Table 1:** Participant age and locale at initial enrollment and at 2.5 ± 0.14 years follow-up.

Parameter	Number (%)	Age Mean (s.d.) Median
Number of subjects who enrolled and provided at least 1 biomonitor sample	97	56.6 (18.5) 58
Females	63 (64.9)	57.6 (17.1) 58
Males	34 (35.1)	55.1 (20.9) 60
Died (as of July 2011) all females	3	85.0 (6.6) 86
Number of subjects who returned follow-up questionnaire at 2.50 ± 0.14 years	73	57.1 (17.9) 59
Females	48 (65.8)	56.8 (16.6) 58
Males	25 (34.2)	57.7 (20.4) 61
States (all subjects with ≥1 biomonitor sample)	97	
Alabama	5 (5.2)	67.2 (17.3) 67
Florida	41 (42.3)	59.6 (17.0) 61
Georgia	36 (37.1)	55.1 (15.2) 55.5
North Carolina	7 (7.2)	30.1 (25.0) 34
Tennessee	8 (8.2)	64.0 (16.0) 57.5
States (subjects remaining at 2.5 years follow-up)	73	
Alabama	4 (5.5)	61.3 (12.7) 65
Florida	32 (43.8)	61.3 (16.1) 62
Georgia	23 (31.5)	57.5 (12.8) 56
North Carolina	7 (9.6)	30.1 (25.0) 34
Tennessee	7 (9.6)	60.9 (14.4) 53

### 2.2. Sample Collection and Preparation

#### 2.2.1. Nail Samples

By our initial protocol, the nail-clipping samples were to be self-collected on a monthly schedule if possible. The subjects were instructed to remove nail polish prior to bathing or showering. Then thoroughly dry the nails, remove any visible debris by scraping, and obtain a clipping from as many TNs as possible using the clippers or scissors they normally use for that purpose. The nail-clippings were placed in the small, pre-labeled paper envelope provided to which the collection date was added by the subject. The samples were mailed to the University of Missouri Research Reactor Center using the pre-addressed, postage-paid return envelopes provided. On receipt, three replicates, in the range of 1 to 25 mg each, were prepared by gravimetrically transferring the clippings to pre-cleaned 0.4 mL high-density polyethylene (HDPE) vials. The clippings were fixed at the bottom of the vial with a pre-cleaned polyethylene plug and filter-paper disk, and closed with a friction-fit HDPE lid.

This sample collection and preparation protocol was followed for those subjects who had retained their TNs throughout the study and when new growth nails became available for those subjects who had lost some or all of their nails. Based on the significant fraction of subjects who had already lost their nails or were in the process of losing them, we modified the sample collection protocol to include FNs and any onycholysitic nail fragments (TN and FN) that subjects had not discarded or those that were subsequently produced. In some cases, nails did not grow out to form a normal hyponychium but instead grew to a length of only a few millimeters (mm) and then spontaneously detached. Onycholysitic nail fragments, varying in length from a few millimeters to nearly the full length of the nail were received. These were subdivided following the distal arc to produce approximate 1.5 mm segments, which were gravimetrically transferred to the HDPE vials as previously described.

Prior to the start of the SSIS, two male subjects had lost all 10 FN samples and retained the onycholysitic fragments along with the dates the fragments detached. These samples were also subdivided for analysis as previously described. One of these subjects has continued to provide both new-growth FN and TN clippings on at least a monthly basis extending his follow-up to over 4 years post exposure.

#### 2.2.2. TBF Product Sample Analysis

In response to our request and provision of sampling materials and a postage-paid mailer, 52 stored samples of the misformulated TBF products and 2 TBF product samples manufactured prior to the Se toxicity outbreak were submitted for analysis including multiple samples from all 6 TBF product lots subject to recall (4016801, 4016802, 4024801, 4031801, 4031802, 4031803). Of these, 36 were accompanied with lot numbers. For 16 TBF samples, the lot number stickers had come off and were discarded by the subject or were not legible.

##### 2.2.2.1. Total Selenium

Each sample was thoroughly mixed, as received, using a vortex shaker and an aliquot was immediately gravimetrically transferred into high-purity (18 MΩ·cm) water to produce a precisely-known dilution factor of approximately 100. These diluted samples were shaken and aliquots were gravimetrically transferred to 0.4 mL HDPE vials containing filter paper to absorb and evenly distribute the liquid samples. The absorbed sample was fixed at the bottom of the vials with an expanded polyethylene plug and closed with a friction-fit HDPE lid. For 50 of the 54 TBF samples, five replicate subsamples were prepared and analyzed. For the remaining 4 samples, 4 replicates were analyzed from 2 samples and 3 and 2 replicates were analyzed from the other 2 samples.

##### 2.2.2.2. Dissolved Selenium

Representative TBF product samples from the diluted stocks were filtered through 0.2 μm filters and replicates from the filtrate were prepared as described above to determine the fraction of Se in a soluble form.

##### 2.2.2.3. Bound Selenium

Ten 0.5 mL aliquots of representative TBF product samples that had been diluted and filtered were transferred to dialysis membrane bags having a 12,000 to 14,000 Da MW cutoff and dialyzed against 18 MΩ·cm water (water:sample = 4000:1) for 18 to 24 h. The dialyzed TBF product replicates were recovered and freeze dried in the membrane bags, which were then transferred to 0.4 mL HDPE vials, fixed and closed as previously described, and analyzed to quantify that fraction of Se associated specifically, or non-specifically, with large molecules.

### 2.3. Neutron Activation Analysis

Se was quantified in the nail and TBF product samples by neutron activation analysis (NAA) at the University of Missouri Research Reactor (MURR) Center using previously described methods [16,17,48,49]. Briefly, samples prepared in HDPE vials, as discussed in previous sections, are irradiated for seven seconds at a thermal neutron flux of 6.5 × 10^13^ n/cm^2^/s using the pneumatic-tube irradiation facility at the MURR Center. Selenium-77m (half life = 17.4 s), produced by neutron capture of stable Se-76, decays by isomeric transition emitting a 161.9 keV photon that is quantified following a 15 s post-irradiation decay by high-resolution gamma-ray spectroscopy using a 30 s real-time measurement period. Pulse pile-up is compensated by the Westphal loss-free counting method and the Se concentration is determined by standard comparison.

### 2.4. Follow-Up Questionnaire

In August 2010, a follow-up questionnaire inquiring about persistent symptoms and lifestyle changes, a cover letter, and a pre-addressed postage-paid return envelope were mailed to the 94 subjects enrolled in the SSIS (Three of the original 97 subjects had died prior to August 2010). Seventy-three (75.3%) completed and returned the questionnaire. The mean ± s.d. follow-up period calculated from the questionnaire completion date and the date of last use of a misformulated TBF product was 2.50 ± 0.14 years. The follow-up questionnaire was organized in four parts. Part 1 asked about any changes to hair and nails. Part 2 collected data on 25 other potential selenosis symptoms. Part 3 asked about changes to how the subject perceived his or her general health relative to their pre-exposure status, occupational impacts, and whether or not they were still under the care of a physician. In Part 4, subjects were invited to provide any additional comments regarding symptoms or quality of life changes that they attributed to their Se intoxication.

### 2.5. Statistical Analyses

Single factor analysis of variance (ANOVA) was used to test for differences in exposure (number of doses), onycholysitic nails (number of nails lost), and extent of adverse health impact (number of symptoms) among the three participant selected groups categorizing the fate of their symptoms at 2.50 ± 0.14 years as improving, the same, or getting worse. Linear regression analysis was used to test for correlations between the peak and restored-baseline TNSe concentrations with exposure (total Se consumed from the TBF products); and the lapsed days between last exposure and onycholysis with the peak FNSe in the onycholysitic fragments. The F-Test was used to test for equality of variances in association with the Student’s *t*-Test to test for differences in the peak TNSe concentration measured in monthly clippings compared to onycholysitic fragments; differences in peak Se concentrations in onycholysitic TN *vs.* FN fragments; and differences in Se concentrations in onycholysitic FN fragments in two subjects, with different exposures, who had identified by digit and saved all 10 FN fragments. All statistical analyses were done using the analysis package in Microsoft Excel 2010.

## 3. Results

A total of 97 subjects from 5 states with a median age of 58 years enrolled in the study and submitted at least one biologic monitor test sample. As of July 2011, three female subjects, with serious preexisting medical conditions had died. However, the influence, if any, their use of the misformulated TBF products had on the cause of death is unknown. Subject data at initial enrollment and again at 2.5 years follow-up are summarized in Table 1. Results including Se concentrations in TBF supplement products, extent of Se overexposure from these products, the temporal response in the TN and FN biologic monitors, and selenosis symptoms experienced and their persistence are reported in the following sections.

### 3.1. TBF Supplement Products and Selenium Exposure

Fifty-four TBF product samples were received and analyzed for total Se by instrumental NAA. Se concentrations expressed as micrograms per 1-ounce dose and ordered by TBF lot number are given in Table 2.

**Table 2:** Se content (μg) per 1-ounce recommended daily dose of Total Body Formula (TBF) dietary supplement products grouped by product lot number.

		Se Content (μg) Per 1-Ounce Dose ^1^				Se Content (μg/Dose) by Lot			
Sample ID	Lot # ^2^^,^^3^	*n*	average	s.d.	%rsdm	*n*	average	s.d.	%rsdm
1	4016801	5	32,397	853	2.63	2	32,184	301	0.93
2	4016801	5	31,972	318	0.99				
3	4016802	5	25,177	181	0.72	7	25,278	111	0.44
4	4016802	5	25,209	165	0.65				
5	4016802	5	25,225	345	1.37				
6	4016802	5	25,263	514	2.04				
7	4016802	5	25,343	482	1.90				
8	4016802	5	25,230	127	0.50				
9	4016802	5	25,501	248	0.97				
10	4024801	5	24,383	236	0.97	3	24,748	325	1.31
11	4024801	5	25,006	299	1.20				
12	4024801	5	24,857	328	1.32				
13	4031801	5	26,090	264	1.01	7	25,850	164	0.64
14	4031801	5	25,667	184	0.72				
15	4031801	5	25,944	133	0.51				
16	4031801	5	25,810	258	1.00				
17	4031801	5	25,624	326	1.27				
18	4031801	5	25,942	452	1.74				
19	4031801	5	25,874	162	0.63				
20	4031802	5	22,490	122	0.54	12	22,319	151	0.68
21	4031802	5	22,489	232	1.03				
22	4031802	5	22,431	201	0.90				
23	4031802	5	22,027	226	1.03				
24	4031802	5	22,194	259	1.17				
25	4031802	5	22,242	414	1.86				
26	4031802	5	22,337	214	0.96				
27	4031802	4	22,187	215	0.97				
28	4031802	3	22,388	91	0.41				
29	4031802	5	22,447	286	1.28				
30	4031802	5	22,170	652	2.94				
31	4031802	5	22,427	196	0.88				
32	4031803	2	25,890	258	1.00	5	25,983	105	0.40
33	4031803	5	25,880	327	1.26				
34	4031803	5	25,975	311	1.20				
35	4031803	5	26,042	119	0.46				
36	4031803	5	26,129	194	0.74				
37	Q1	5	22,249	203	0.91				
38	Q2	5	22,384	173	0.77				
39	Q3	5	25,452	201	0.79				
40	Q4	5	25,628	224	0.87				
41	Q5	5	25,896	287	1.11				
42	Q6	5	26,172	280	1.07				
43	Q7	5	26,234	228	0.87				
44	Q8	5	26,252	279	1.06				
45	Q9	5	32,302	165	0.51				
46	Q10	4	32,872	302	0.92				
47	Q11	5	32,899	165	0.5				
48	Q12	5	33,025	197	0.6				
49	Q13	5	33,084	342	1.03				
50	Q14	5	33,128	138	0.42				
51	Q15	5	33,221	392	1.18				
52	Q16	5	33,894	122	0.36				
53	Q17	5	911	16	1.76				
54	Q18	5	234	14	6.14				

^1^ Each liquid supplement sample was analyzed in replicate and reported as the average and standard deviation based on the Se content per the one-ounce recommended daily dose. ^2^ Lot numbers beginning with “Q” reference those supplement product samples from which the lot number label was lost or not legible. ^3^ Lot numbers Q17 & Q18 are TBF products manufactured prior to the misformulated lots (September 2007).

Based on the certificate of analysis of the vitamin-mineral dry mixture, Se was included as sodium selenate (Na_2_SeO_4_) at a concentration of 112.5 mg Se per 1-ounce dose. At this concentration, sodium selenate is completely soluble in water even at 0 °C. The TBF product samples received from SSIS participants contained some suspended solids that did not separate under refrigerator storage. To demonstrate that the Se was in solution and not associated with the suspended solids, we filtered a sample through a 0.2 μm membrane and analyzed replicates of the filtrate. Our recovery of Se in the filtrate was 99.4% ± 2.1% indicating, within measurement error, that all the Se was in solution.

To demonstrate that the Se was not specifically or non-specifically bound to a large molecule, we dialyzed TBF samples against 18 MΩ·cm water using bags fashioned from dialysis tubing having a 12,000 to 14,000 Da MWCO. The dialyzable component contained 98.0% ± 0.7% of the Se, which is consistent with dissolved sodium selenate.

**Figure 1:**
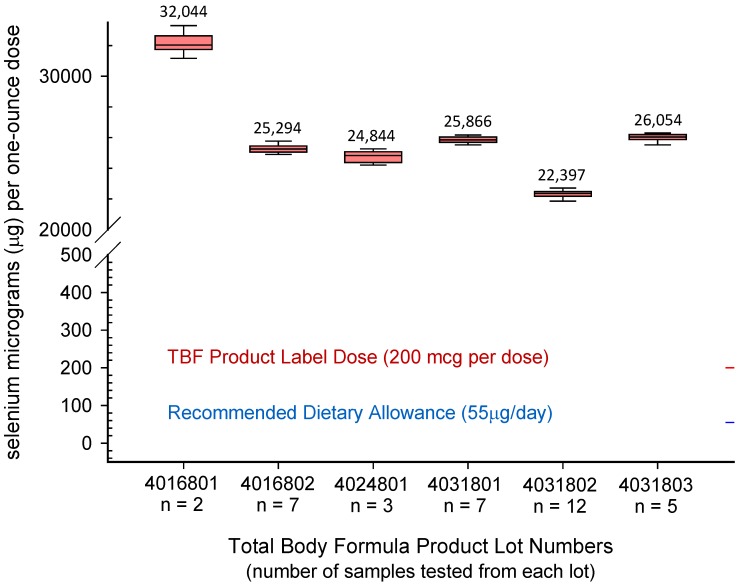
*Se concentrations (μg/dose) in the six recalled TBF product lots.* Se concentrations are given in micrograms per 1-ounce recommended dose for the 36 (of 54) samples submitted with corresponding lot numbers. The dose means (s.d.) range from 22,319 (151) to 32,184 (301) μg/dose over the 6 lots. The intended dose for all six lots was 200 μg/dose. Each box plot indexes the 10th, 25th, 50th (median), 75th, and 90th percentiles. An additional 16 samples without lot numbers, but obviously distributed among the 6 recalled lots, and 2 samples from TBF lots produced prior to September 2007, were also submitted and analyzed. Results for all 54 samples are summarized in Table 2.

There was substantial variation in Se content among the 6 lots ranging from lot means of 22,319 ± 151 to 32,184 ± 301. The Se concentration distributions within and between the 6 misformulated TBF lots are shown as a box plot in Figure 1.

Sixty-seven subjects reported the number of doses of TBF they consumed, which ranged from 5 to 64 with a mean of 30.9 ± 13.9 and a median of 30. With few exceptions, subjects reported using the supplement on a daily basis. This is in good agreement with that reported in an earlier study [47] for 156 subjects who consumed 1 to 109 doses with a median of 29. For the individual consuming the median 30 doses, the total Se consumed, depending on the lot number, was 669,570 to 965,520 μg of Se compared to the RDA of 1650 to 2100 μg assuming a 30-day consumption period.

### 3.2. Toenail and Fingernail Results

#### 3.2.1. Temporal Response Measured in Toenail Samples

Toenail and fingernail selenium (FNSe) concentrations (μg/g) for those subjects in which a temporal response could be measured are summarized in Table 3. It was possible to determine the maximum post-exposure TNSe concentration for a total of 30 subjects, 14 who did not lose their TNs and were able to provide clippings on an approximate monthly basis and 16 subjects who submitted onycholysitic fragments that could be subdivided into approximate 1.5 mm segments from which the temporal response was determined. Ten subjects provided onycholysitic FN fragments from which a temporal response could be determined. A total of 45 subjects provided either post-exposure, continuous, monthly clippings or post-exposure, restored, new-growth TN samples from which the restored baseline could be determined. The Se concentration data for these subjects are also summarized in Table 3.

**Table 3:** Summary of toenail and fingernail Se concentrations among subjects with post-exposure peak and restored-baseline measurements ^1^.

	Subject Toenail and Fingernail Samples from Which the Post-Exposure Peak and Restored-Baseline Se Concentration were Measured				
	Peak Concentrations (μg/g)				Restored-Baseline Concentrations
	Toenail Se (μg/g) All Samples	Toenail Se (μg/g) Monthly Samples	Toenail Se (μg/g) Onycholysitic Samples	Fingernail Se (μg/g) Onycholysitic Samples	Toenail Se (μg/g) Monthly Samples
*n*	30	14	16	10	45
Mean ^2^	8.27	4.91	11.17	22.64	0.809
Std. Dev.	4.41	2.69	3.51	8.14	0.125
Median	8.40	4.45	9.70	21.35	0.789
Minimum	1.20	1.20	7.70	13.80	0.610
Maximum	18.30	10.10	18.30	44.10	1.213
TBF dose response (see Figure 2A,B)	*m* = 0.008963				*m* = −0.00015
	*b* = 1.2436				*b* = 0.9049
	*r* = 0.668				*r* = 0.340
	*p* = 0.00006				*p* = 0.022

^1^ Nail Se concentration in normal subject (diet only/no supplementation) is approximately 0.85 μg/g; ^2^ Peak TNSe (onycholysitic fragments) > peak TNSe (monthly clippings) *p* = 9 × 10^−6^; Peak FNSe (onycholysitic fragments) > peak TNSe (onycholysitic fragments) *p* = 1.4 × 10^−3^.

**Figure 2:**
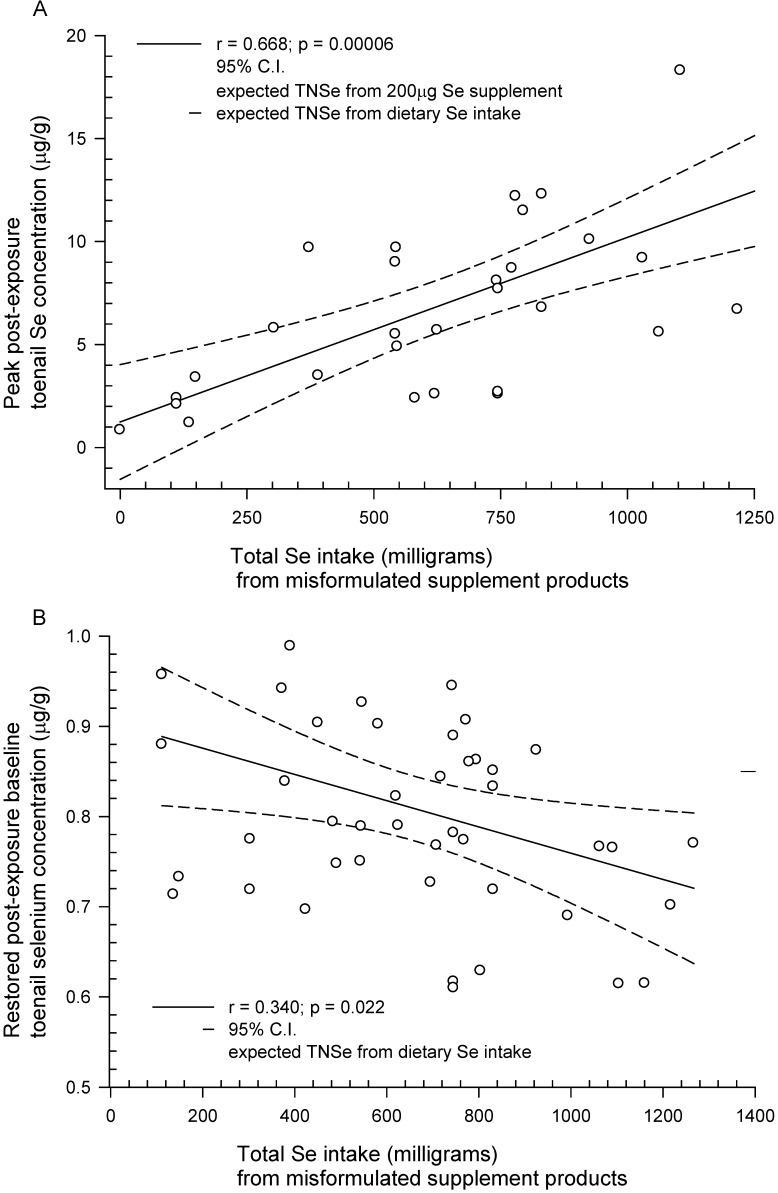
*Dose responses measured in the toenail biologic monitor relating peak (**A**) and restored-baseline (**B**) Se concentrations (μg/g) to total Se exposure (total milligrams Se consumed)*. The peak TNSe concentration (μg/g) could be measured in 29 subjects who submitted dose-consumption information and either monthly samples or onycholysitic nail fragments that could be segmented to give a chronological profile. As expected the peak TNSe concentration was directly correlated with Se exposure (**A**). The negatively-correlated temporal response (**B**) for the restored-baseline TNSe concentration relative to exposure was measured in 45 subjects including the initial group and those subjects who had either joined the study after their TNSe concentration peak had passed or were unable to collect monthly samples in the early phase of the study but were able to submit “new growth” samples as their nail growth was restored.

**Figure 3:**
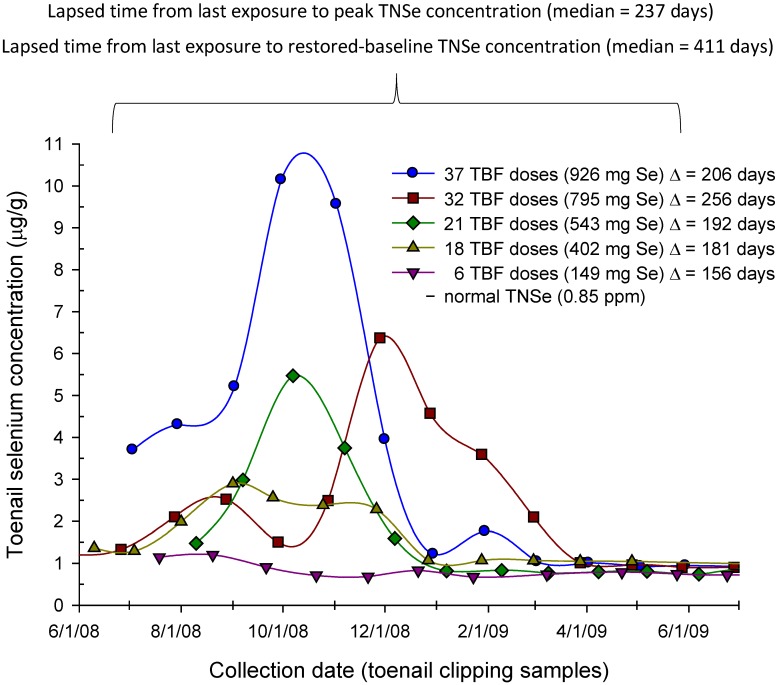
*Temporal response in toenail Se concentration (μg/g) from monthly toenail clipping samples relative to Se exposure (6 to 37 doses; 149 to 926 mg Se) from misformulated TBF supplement products.* The temporal response in toenail Se (TNSe) concentration for 5 representative subjects ranging in Se exposure from 6 to 37 doses (149 to 926 mg Se) who were able to submit monthly samples is somewhat variable in the breadth of the elimination period, the number of peaks, and the lapsed time from last use of the misformulated product to the peak TNSe concentration (Δ days). The peak TNSe concentration is highly correlated with total Se intake from the TBF products (*r* = 0.96, *p* = 0.01) in these 5 subjects. The median lapsed times spanning the last exposure to the TNSe peak concentration and the restored-baseline concentration, were 237 and 411 days, respectively.

The post-exposure peak and baseline-restored Se concentrations were positively and negatively correlated, respectively, with total Se consumed from the misformulated TBF supplement products. The linear slopes (*m*) intercepts (*b*), correlation coefficients (*r*), and *p*-values (*p*) are given in Table 3. The total Se consumed (mg) *vs.* peak and *vs.* baseline-restored TNSe concentrations are shown in Figure 2A,B, respectively. The temporal responses in the TNSe concentration for 5 representative subjects in the monthly-clippings group, and spanning most of the range of doses consumed, are shown in Figure 3.

#### 3.2.2. Temporal Response Measured in Onycholysitic Fingernail Samples

**Figure 4:**
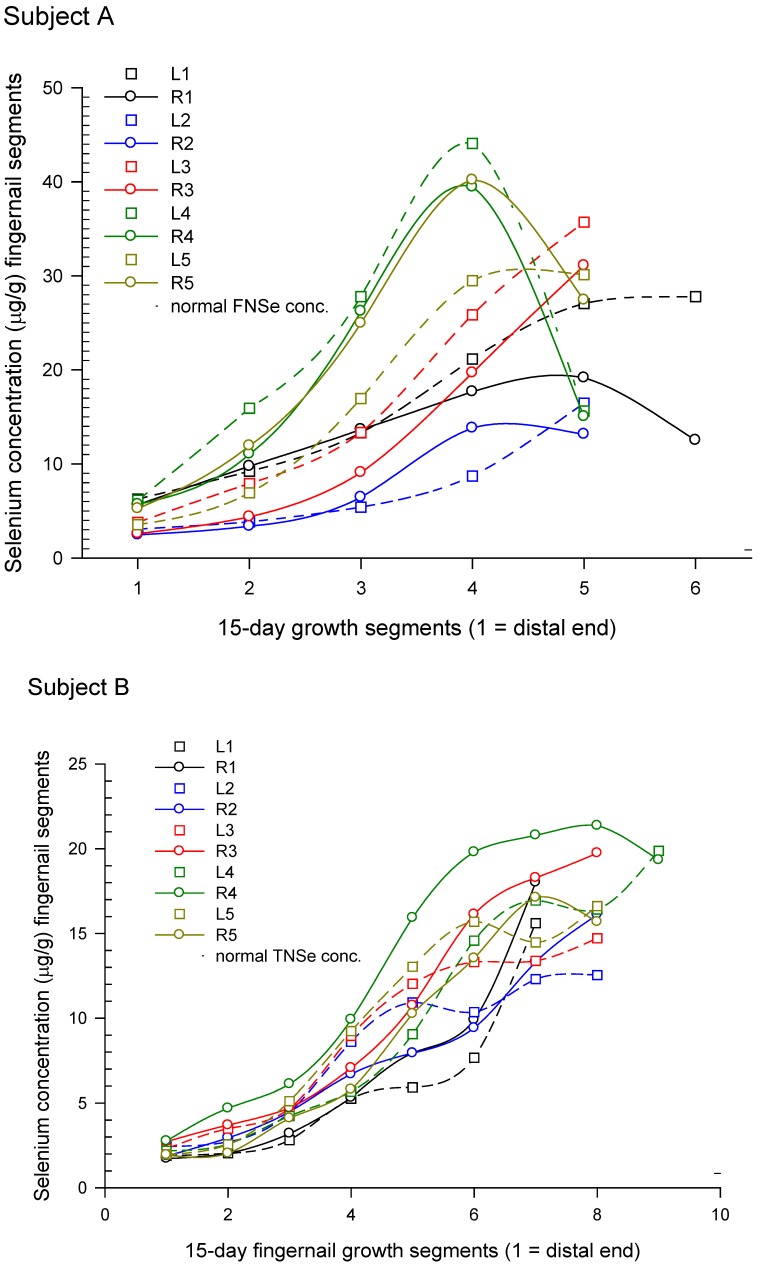
*Temporal response in fingernail Se concentration (μg/g) from segmented onycholysitic fragments from all ten fingernails in two subjects who consumed 44 (Subject **A**) and 32 (Subject **B**) doses of misformulated TBF supplement products*. These two subjects lost fragments from all 10 FNs that were approximately half the length of the entire nail. In the graph legends, the nails are identified as L (left), R (right) and 1 through 5 as thumbnail, index FN, middle FN, ring FN and little FN, respectively. These fragments were subdivided along the arc defined by the distal end of the fragment. Each segment was approximately 1.5 mm and was analyzed separately for Se. Each subject reported the TBF product lot numbers, total number of 1-ounce doses consumed, and the use period. From these data we compute that Subject A consumed 1,104,340 μg of Se over a 50 day period and Subject B consumed 831,456 μg over a 34 day period. The nail Se concentrations for the two subjects reflect their exposures (Subject A > Subject B; *p* = 0.0007). For both subjects, the highest Se concentrations were found in the ring FN (LR4). Then the Se concentrations followed LR5 > LR3 > LR1 > LR2 and LR3 > LR5 > LR1 > LR2 for Subjects A and B, respectively.

The temporal responses, measured individually in onycholysitic fragments from all 10 FNs, for 2 male subjects who saved their detached nails and recorded the dates of detachment, are shown in Figure 4A,B. These two subjects lost fragments from all 10 FNs that were approximately half the length of the entire nail. The lapsed days between the last use of the misformulated TBF products and dates of FN-fragment detachment ranged from 27 to 114 days for the 2 subjects and were negatively correlated with the whole-fragment Se concentrations (*r* = 0.67, *p* = 0.0012).

These fragments were subdivided along the arc defined by the distal end of the fragment. Each segment was approximately 1.5 mm and was analyzed separately for Se. Each subject reported the TBF product lot numbers, total number of 1-ounce doses consumed, and the use period. From these data, Subject A consumed 1,104,340 μg of Se over a 50 day period and Subject B consumed 831,456 μg over a 34 day period. The nail Se concentrations for the two subjects reflect their exposures {[Subject A (fragment mean ± s.d. = 14.2 ± 4.5)] > [Subject B (fragment mean ± s.d. = 7.2 ± 1.9)], *p* = 0.0007}. For both subjects, the highest Se concentrations were found in the ring FNs and the least in the index FNs. The temporal responses from the same FN fragments, left and right, are generally more similar to each other than fragments from other FNs (Figure 4A,B).

**Figure 5:**
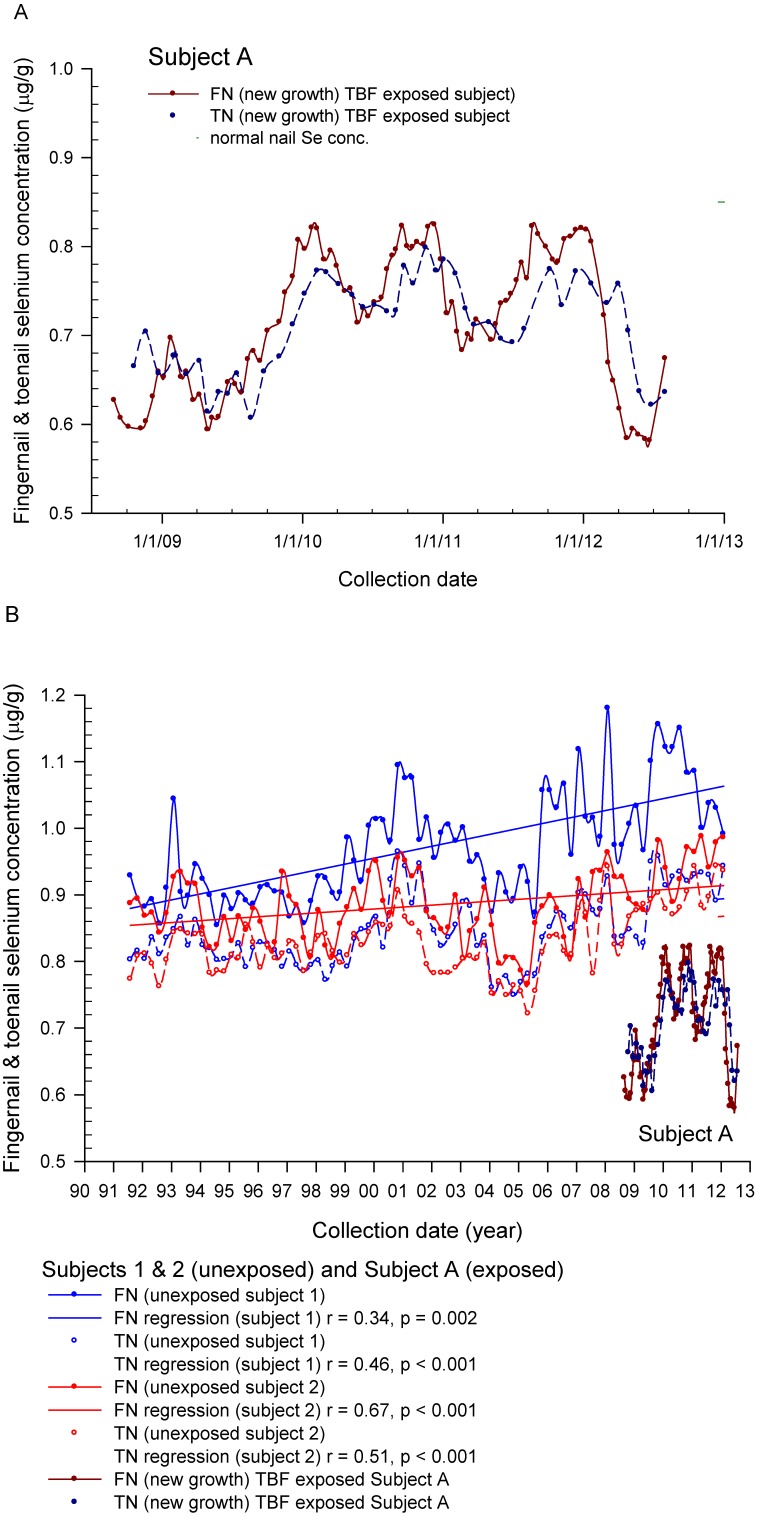
*Post-exposure, post-peak temporal response in new-growth fingernail and toenail Se concentrations (μg/g) in Subject A (**A**) and in comparison to 2 other unexposed male subjects who do not use Se supplements (**B**).* The post-exposure restored baseline for Subject A has been measured in new-growth FN and TN samples collected twice-a-month for FNs and once-a-month for TNs over approximately 4 years. Each FN and TN data point in (A) is the mean of 10 individual FN and TN clipping samples taken individually from each nail and analyzed separately. Done in this way each data point integrates dietary Se intake over an estimated 2 weeks for FNs and 4 weeks for TNs in the context of the temporal response profiles. For both FNs and TNs the post-exposure restored baselines cycle with a near annual period while remaining below the normal Se concentration expected for this subject (0.85 μg/g). As expected, the TN monitor lags somewhat behind the FN monitor. The post-exposure restored baselines for Subject A (male, Caucasian, non-smoker) are compared to 2 male subjects of comparable age, race, diets, and BMI in (**B**). The 2 comparison subjects have, for over 20 years, participated in a Missouri study of Se status in which they provide FN and TN samples on a quarterly schedule. The quarterly FN and TN samples include clippings from all FNs in the FN sample and all TNs in the TN sample. Representative aliquots for analysis are prepared from the combined FN and TN quarterly samples. Neither Caucasian comparison-subject smokes cigarettes or takes a Se supplement. There has been a gradual increase in both FNSe and TNSe concentrations observed in both comparison subjects over the 20-year study period attributable to increasing dietary Se intakes.

The post-exposure restored baseline for Subject A has been measured in new-growth FN and TN samples collected twice-a-month for FNs and once-a-month for TNs over approximately 4 years. Each clipping for each FN and TN was identified and segregated by the subject and then analyzed individually for Se. Chronological profiles for all 10 FNs and TNs were maintained (data not shown). The graphs based on the 10-digit means are shown in Figure 5A. Done in this way each data point integrates dietary Se intake over 2 weeks for FNs and 4 weeks for TNs in the context of the temporal response profiles. For both FNs and TNs the post-exposure restored baselines cycle with a near annual period while remaining below the normal Se concentration expected for this subject (0.85 μg/g). As expected, the TN monitor lags somewhat behind the FN monitor.

The post-exposure restored baselines for Subject A (male, Caucasian, non-smoker) are compared to 2 male subjects of comparable age, race, diets, and BMI in Figure 5B. The 2 comparison subjects have, for over 20 years, participated in a Missouri study of Se status in which they have provided quarterly FN and TN samples. Representative aliquots for analysis are prepared from the combined FN and TN quarterly samples. Neither Caucasian comparison subject smokes cigarettes or takes a Se supplement. There has been a gradual increase in both FN and TN Se concentrations observed in both comparison subjects over the 20-year study period attributable to increasing dietary Se intakes. This is in agreement with a pilot study in which we measured an 8.3% mean increase (*p* = 0.0001) in the TNSe concentrations in samples collected 17 years apart (1987 to 2004) from 53 subjects. Subject A resides in Tennessee and would be expected to have FN and TN baselines that overlap with the 2 comparison subjects residing in Central Missouri.

### 3.3. August 2010 Follow-Up Questionnaire

#### 3.3.1. Selenium Toxicity Symptoms

**Figure 6:**
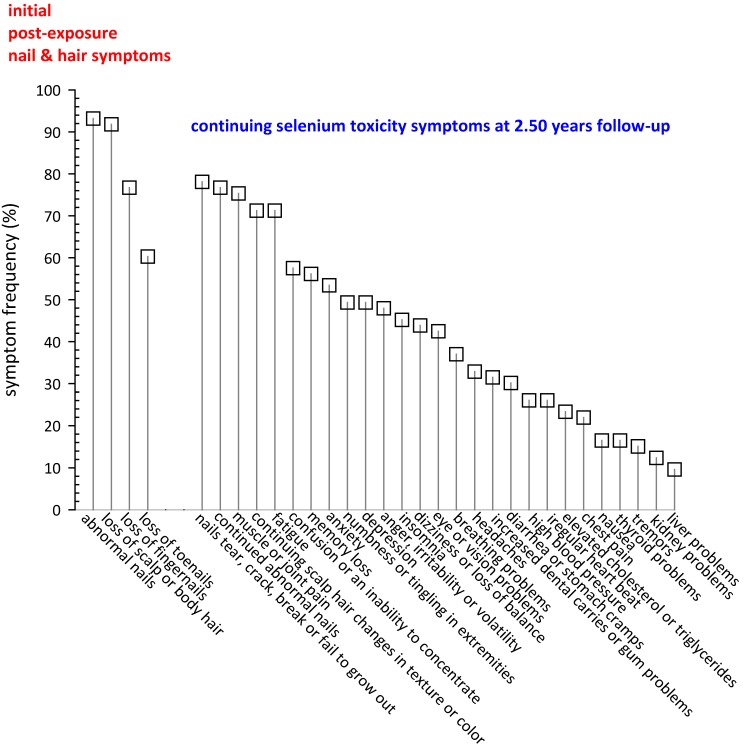
*Initial and continuing symptoms of Se toxicity.* Seventy-three subjects returned an August 2010 questionnaire inquiring about the impact of their consumption of the misformulated TBF supplement products on their hair and nails and their persistent Se toxicity symptoms. At 2.50 ± 0.14 years follow-up, these 73 subjects reported 1 to 27 persistent symptoms they attributed to Se toxicity; mean = 11.5 ± 6.5; median = 10.

At 2.50 ± 0.14 years’ follow-up, seventy-three subjects returned an August 2010 questionnaire inquiring about the impact of their consumption of the misformulated TBF supplement products on their hair and nails and their persistent Se toxicity symptoms. These outcomes are summarized in Figure 6.

Of these 73 subjects, 93.2% initially reported abnormal nails, 76.7% loss of FNs (mean = 9.6 ± 1.6; median = 10 FNs), 60.3% loss of TNs (mean = 6.5 ± 3.7; median = 8 TNs), and 91.8% loss of scalp or body hair. Respondents reported 1 to 27 persistent symptoms (mean = 11.5 ± 6.5; median = 10 symptoms) that they attributed to their use of the misformulated TBF products and the resulting Se intoxication. Other than hair and nails, the most frequently reported persistent symptoms were muscle or joint pain (75.3%), fatigue (71.2%), and numerous adverse neurological symptoms (~50%). For more detail, see Figure 6. Sixty-three subjects responded to the categorical question regarding disposition of symptoms at 2.5 years with 36 (57.1%) reporting symptoms improving, 21 (33.3%) symptoms the same, and 6 (9.5%) symptoms getting worse. Subjects reporting improving symptoms also reported fewer symptoms (9.9 ± 4.8) compared to the subjects reporting their symptoms to be about the same (14.2 ± 6.0; *p* < 0.005) and compared to the subjects reporting their symptoms getting worse (21.3 ± 4.7; *p* < 3 × 10^−6^). The increase in number of symptoms in the “same” to “worse” groups was also statistically significant (*p* = 0.013). Thirty-four subjects (46.6%) responded that they remained under a physician’s care for symptoms they attributed to Se toxicity at 2.5 years post exposure.

Seventy-two subjects reported both the number of TNs and FNs lost (range TN + FN = 0 to 20) and also number of symptoms (range = 1 to 27). In a correlation analysis, the TN + FN sum is a good predictor of number of symptoms (directly correlated; *r* = 0.42; *p* = 0.00025). Similarly, the sum of TN + FN lost increases in those fate-of-symptoms groups in which the subjects have self-assigned themselves from symptoms improving (TN + FN = 10.2 ± 7.6 nails lost) to symptoms the same (TN + FN = 11.5 ± 6.9 nails lost) to symptoms getting worse (TN + FN = 17.7 ± 3.6 nails lost). Of these categories, the symptoms worse group is significantly different from the symptoms improving group (*p* = 0.023) and the symptoms the same group (*p* = 0.05) but the symptoms improving and the symptoms the same groups are not different (*p* = 0.53).

#### 3.3.2. Occupational Impact

Thirty-eight of 67 respondents indicated that they were employed outside the home or were actively seeking employment coincident with their use of the misformulated TBF products. Of these thirty-eight, 25 (65.8%) reported that their employment was negatively impacted by their Se toxicity symptoms. Some were fired, some quit their jobs, some retired, some had their employment status downgraded, some reduced their hours, some took extended sick leave, and some suspended their job search activities. The most frequently cited job-related problems were fatigue, particularly in physically challenging jobs, and lack of concentration, particularly in detail-oriented jobs.

## 4. Discussion

### 4.1. Other Studies of Selenium Toxicity

#### 4.1.1. Cases of Accidental and Intentional Selenium Poisoning

In addition to Se intoxication from misformulated dietary supplements [18,19,20], Brazil nuts are cited as a food source having high Se and have the potential to cause Se toxicity [50,51,52,53,54]. There have also been several reports covering serious injury and deaths from environmental, occupational, accidental, suicidal, and homicidal Se poisoning [55,56,57,58,59,60,61]. These reports provide important information on the severely acute symptoms associated with lethal Se poisoning and also on the corresponding Se concentrations found in body fluids and the most critically implicated tissues.

#### 4.1.2. Selenium Toxicity from Misformulated TBF Products—Previous Studies

To date there have been 5 reports published regarding the TBF Se toxicity outbreak [47,62,63,64,65] and an invited commentary reflecting on the need for congress to revisit the 1994 Dietary Supplement Health and Education Act (DSHEA) citing the inadequacy of the Act to protect the general public from the TBF Se toxicity outbreak [66]. 

In June 2008, a report [62] on a 55 year-old female subject who had used a misformulated TBF product listed her selenosis symptoms as “diarrhea, hair loss, nail abnormalities, muscle cramps, joint pain and impaired mental concentration. Serum and urine Se concentrations were elevated at 534 μg/L and 220 μg/L, respectively. Details were not given regarding this subject’s total exposure or the delay times between last use of the TBF product and onset of symptoms or blood and urine sampling. No follow-up of symptoms or biomonitor Se concentrations were given.

In a December 2008 report on 63 Georgia subjects who consumed the TBF products [63], selenosis symptoms including “fatigue, hair loss, joint pain, muscle pain, nail abnormalities, headache, nausea, foul breath, rash, vomiting, fever, diarrhea and limb control” were compared at 2 time points, April and June 2008. Improvement was observed for most symptoms. No details of exposure or Se concentrations in biomonitor samples were given.

In a February 2010 report [47], 201 subjects from 9 states who had consumed the misformulated TBF products over a median period of 29 days were comprehensively discussed. Data on selenosis symptoms were collected at 2 time points; initially, within a short time following the presentation of selenosis symptoms, and again at least 90 days after subjects’ last use of the TBF products. Symptoms identified in the initial survey in order of decreasing frequency were: “diarrhea, fatigue, hair loss, joint pain, nail discoloration/brittleness, nausea, headache, tingling, vomiting, fever and ataxia”. Persistence of symptoms at 90+ days post exposure ranged from 11% to 52% with nail discoloration, brittleness or loss being the most frequently reported (52%). Urine Se was measured in 7 subjects (median = 179 μg/day) and serum Se in 9 subjects (median = 664 μg/L). Both of these metrics were elevated and as expected, variable, depending on duration between exposure and measurement. Urine and serum Se returned to normal levels at 1 to 2 weeks and 6 weeks, respectively, post exposure.

In a July 2010 letter to the editor [64] 2 subjects were briefly discussed. Symptoms reported included hair loss, nail abnormality, dizziness, fatigue, amenorrhea and dental caries. In one of the subjects, serum and urine Se levels were in the normal range when measured 5 weeks post exposure. A hair sample had an elevated Se concentration at 3.2 μg/g.

In a January 2012 case series report [65], 9 subjects who had consumed the misformulated TBF products for a mean period of 37.5 days were discussed. Initial symptoms, which began within 1 week of ingestion, were listed as “alopecia, dystrophic FN changes, GI symptoms, and memory difficulties”. Whole blood Se concentrations from samples taken on average at 27 days post exposure ranged from 150 to 732 μg/L and urine Se concentrations ranged from 41 to 220 μg/g creatinine.

### 4.2. TBF Supplement Products and Selenium Exposure—This Study

Our analyses of the TBF products found Se concentrations in the 6 misformulated TBF product lots ranged from 22,319 ± 151 to 32,184 ± 301 μg per 1-ounce dose (μg/dose). All of the Se is soluble (99.4% ± 2.1%) and inorganic (98.0% ± 0.7%) consistent with the sodium selenate formulation reported by the company that produced the dry vitamin-mineral mixture. Our TBF product analyses of Se concentrations were lower than that calculated from the Certificate of Analysis (COA) for the vitamin-mineral dry mixture, 47,000 μg/dose, and that reported by the FDA 40,800 μg/dose [44]. Efforts to determine details of the FDA analyses (methodology, lot numbers, number of samples) were unsuccessful. Our results are in good agreement with the 24,015 μg/dose previously reported for a single TBF product sample of unknown lot number [61]. We analyzed multiple replicates (generally 5) of 52 samples of misformulated TBF products including multiple samples (2 to 12) from all 6 lots. See Table 2. Our results were consistent for samples in the same lot; exhibited good precision among replicates for any given sample; and were in excellent agreement with certified or accepted values for the quality control samples (data not shown) analyzed along with the TBF samples. Questionable practices including an unjustified and erroneous alteration of the COA (as described in the court records) of the dry vitamin-mineral mixture and the absence of any laboratory analysis of the dry mixture for Se argue that confidence in Se concentrations derived from manufacturing data is not warranted. In addition, the variation in Se content over the 6 misformulated lots, all having an intended concentration of 200 μg/dose, further demonstrates the poor quality control under which the TBF products were produced (Figure 1). While there is no evidence that TBF products manufactured prior to September 2007 contained the highly elevated Se concentrations observed in the instant case, our analysis of two such products found Se concentrations of 234 ± 14 and 911 ± 16 μg per 1-ounce dose compared to a label value of 200 μg/dose. The modest overage (17%) we found in the 234 μg/dose product is similar to what we have measured in other dietary supplements containing Se [17]; however the 356% overage measured in the 911 μg/dose product substantially exceeds anything we have measured previously but is itself dwarfed by the 6 misformulated TBF lots produced in September 2007. This suggests that control of the Se concentration in TBF products may have been a long-standing problem prior to 2007.

The Se exposures from the misformulated TBF products over the median one-month use period ranged from 669,570 to 965,520 μg depending on the TBF lot compared to 1650 to 2100 based on the RDA, 12,000 based on the UL, and 27,330 based on the LOAEL. Furthermore, unlike a previous Se toxicity outbreak caused by a misformulated supplement containing a similar concentration of Se but in a solid dosage form [18,19], the TBF products contained Se in which at least 98% was completely solubilized in water requiring no digestion and hence readily bioavailable. To our knowledge, the Se toxicity outbreak caused by the misformulated TBF products exceeds that of any other resulting from a dietary supplement in terms of the median quantity of Se ingested and severity of the adverse health effects the victims suffered.

### 4.3. Toenail and Fingernail Biologic Monitors of Selenium Status

The use of TNs and FNs to estimate Se intake and as a surrogate of Se status in epidemiological studies is well established [12,48,49,67,68,69,70,71,72,73,74,75,76,77,78,79,80,81]. Collectively these papers demonstrate that the nail Se concentration reflects integrated intake over several weeks and is retrospective back 3 to 18 months [12,72,73]; is reproducible over several years [69,74]; is sensitive to determinants such as supplement use, cigarette smoking, race, alcohol consumption and geographic location [16,48,67,68,70,71,77,81]; and can be accurately and feasibly measured by instrumental neutron activation analysis in large cohort studies [42,49,75,76,78,79,80]. Se has been shown to be incorporated in keratinacous protein (hair and presumably nails) largely as selenocystine (91% ± 4%) by near-edge X-ray absorption spectroscopy [82]. Finally, TNSe concentrations have been demonstrated to be directly correlated to Se concentrations of critical organs in both animal [83] and human [84] models.

We have measured TNSe concentrations in 29,552 adult subjects in the U.S. including males and females with substantial numbers of Caucasians, African Americans and others and covering the entire country with the exception of Alaska. Excluding 27 samples having a TNSe concentration greater than 2 μg/g, the overall mean ± s.d. is 0.92 ± 0.30 μg/g and concentrations, in the 10th, 25th, 50th, 75th and 90th percentiles, are 0.72, 0.79, 0.88, 0.99 and 1.11 μg/g, respectively. Other than dietary intake, Se supplement use (positive) and cigarette smoking (negative) are the major determinants of TNSe concentration, which follow Caucasians > Others > African Americans. TNSe concentration more-or-less follows the soil Se concentration with higher concentrations in the northwest and lower concentrations in the southeast where most of the Se toxicity cases occurred in the outbreak caused by the misformulated TBF products. From our data, we estimate that the normal TNSe concentration in the TBF impacted area to be 0.85 μg/g.

In the SSIS, the peak TNSe and FNSe concentrations in the exposed subjects are 13 and 27 times higher, respectively, compared to subjects consuming Se at physiological levels, such as the estimated 60 to 220 μg per day in the U.S. [21,22,23,24,25] where TNSe concentrations are only modestly lower than corresponding FNSe concentrations. For example, in Subject A the peak FNSe concentration measured in an onycholysitic FN fragment (44.1 μg/g) is 2.4 times higher than his peak TNSe concentration (18.3 μg/g) also measured in an onycholysitic fragment (see Table 3). However, in Subject A’s restored baseline the FNSe-to-TNSe ratio is only slightly greater than 1 averaged over an extended period (Figure 5A).

As expected, both TNSe and FNSe concentrations reflect exposure (Figure 2A, Figure 3, Figure 4). In those cases where there is a regular (for example, daily) sub-lethal acute selenium exposure, the cumulative Se intake can be estimated from the peak FNSe or TNSe concentrations measured in monthly nail clippings or one or more onycholysitic fragments. The retrospective nature of the nail biologic monitor for Se makes possible the measurement of the temporal response including the rise to the peak, the peak, and the return to baseline (Figure 3) well after the exposure has occurred. This is not feasible in short-term monitors such as blood and urine, and substantially more difficult using hair due to alopecia and the sampling challenges imposed. The post-exposure, post-peak return to baseline is negatively correlated with exposure in the SSIS subjects (Figure 2B) suggesting that in the more extreme Se toxicity instances one or more determinants of Se homeostasis is perturbed. This presumed lower Se status can persist for years post-exposure as illustrated in Figure 5A,B. The temporal response to acute Se toxicity can also be measured using segmented onycholysitic FN fragments as illustrated for 2 subjects in Figure 4A,B. Interestingly there is a substantial variation in the peak FNSe concentration among the different FNs. In both subjects, the highest and lowest FNSe concentrations appear in the ring and index FNs, respectively. The relative standard deviations of the means (rsdm) in the FNSe peak segments in these two acutely Se-exposed subjects are 35.2% (Subject 4A) and 15.6% (Subject 4B) compared to 4.89% measured in 87 ten-digit FN-clipping samples collected during the restored-baseline period for Subject 4A.

### 4.4. Persistence of Selenium Toxicity Symptoms in the Selenium Supplement Intoxication Study (SSIS)

The selenosis symptoms reported by the SSIS subjects generally parallel those reported in previously published studies of the TBF Se toxicity outbreak [61,62,63,64,65]; other reports of Se toxicity caused by misformulated supplements [18,19,20]; Se toxicity caused by over-indulgence in Brazil nuts [50,51,52,53,54]; and cases of environmental, occupational, accidental, suicidal, and homicidal Se poisoning [55,56,57,58,59,60,61]. However, a major difference is that these studies do not report long-term follow-up. To the extent they address the consequences associated with non-lethal Se toxicity, the impression left is that Se-intoxicated subjects recover their previous health after some period during which their adverse hair and nail abnormalities are for the most part reversed. This is not the finding in the SSIS where 42.8% of the subjects reported that their symptoms were the same or were getting worse at 2.50 ± 0.14 years after their last exposure; and a similar fraction (46.6%) remained under the care of a physician for symptoms the subjects attribute to their Se toxicity. We believe this disparity in the SSIS findings compared with other reports largely results from a lack of systematic follow-up in these studies caused by the infrequency at which acute Se toxicity outbreaks and poisonings occur; the generally small number of individuals exposed in any single outbreak; the non-specificity of many selenosis symptoms; the absence of timely diagnostic testing to detect the existence and magnitude of a Se toxicity outbreak; and the absence of effective Se toxicity treatments other than supportive care to reduce the adverse impact of the symptoms. The SSIS has in part overcome some of these limitations and finds that many serious selenosis symptoms such as muscle and joint pain, fatigue, neurological problems, respiratory problems, and in some cases, thyroid and kidney failure, persist long term and may be permanent disabilities.

### 4.5. Limitations in the SSIS

The SSIS was undertaken in response to an unintended exposure of users of misformulated TBF supplement products resulting in acutely toxic Se intakes. The misformulated products were on the market and in use for almost 3 months before publication of the first FDA news release identifying them as the cause of a 10-state Se toxicity outbreak [43]. The SSIS protocol required IRB approval prior to contacting potential participants and enrolling those interested in the study. This delay limited the number of subjects for whom a full-spectrum temporal response in the FN and TN biologic monitors could be measured. The measurement of the temporal response was further complicated by the severity of the Se toxicity on FN and TN physiology. Many victims suffered onycholysis losing large nail fragments prior to learning the cause. In most cases the fragments were discarded prior to the victims being advised to retain them for analysis. However, even with this limitation, a full-spectrum temporal response of the TNSe concentration was measured in approximately 15% of the population estimated to have been significantly exposed in the outbreak.

Time and resources have limited the SSIS to self-reported selenosis symptoms, their persistence, and adverse impacts on occupations, lifestyles and quality of life. Hence we have not independently verified the self-reported data or been able to consider pre-existing conditions. Lack of resources has also limited a more robust long-term follow-up in the SSIS. For example, the impact of this acute toxicity on the selenoproteome, and particularly selenoenzyme activities, would provide useful information.

## 5. Conclusions and Implications

At least 201 persons are estimated to have been exposed to acutely toxic levels of Se from their use of the misformulated TBF products. Of these, Se concentrations in blood and urine were measured in other studies in fewer than 10 percent of exposed subjects; and in no case was a temporal response measured in which the peak Se concentration in blood or urine could be determined. In the SSIS we were able to measure a full-spectrum temporal response using the toenail biologic monitor in approximately 15% of the exposed subjects and the post-exposure return to baseline in over 20%. We were able to demonstrate that both nail clippings and onycholysitic nail fragments from both TNs and FNs accurately quantify Se exposure. Health professionals should incorporate these diagnostic biologic monitors when confronted with the possibility of Se poisoning. Patients should be instructed to collect and save FN and TN clippings, identified by collection date, over the next 12 months; and retain, identify, and date any onycholysitic FN and TN fragments that may spontaneously detach during that period. The peak FNSe or TNSe concentration can be used to estimate the cumulative Se exposure, the likely selenosis symptoms, and their persistence.

The median Se exposure, in the form of solubilized inorganic selenate, in the SSIS ranged from 669,570 to 965,520 μg over an approximate 30 day period, which exceeds the RDA for Se by a factor of 300 to 585 leading to selenosis symptoms and adverse health effects, some debilitating, that persist 2.5 years subsequent to the last exposure to the misformulated TBF products in 43% of the subjects studied. This finding is contrary to the suggestion in the literature that people who have suffered non-lethal acute Se poisoning would fully recover within the SSIS follow-up period of 2.5 years. For Se, the lethal one-time dose is not precisely known but has been reported to be 4 mg per kilogram in “untreated animals” [57], which, for a 70 kilogram adult would be 280,000 μg. This quantity of Se would be consumed in 9 to 13 doses of the misformulated TBF products. Inorganic Se metabolism, as selenite, has been studied in the human at physiological levels of intake using an enriched stable Se-74 tracer [85]. Se is metabolized into 4 plasma compartments, a compartment comprised of the liver and pancreas, and a tissue pool that turns over slowly with a retention time of 115–285 days accounting for approximately 35% of the absorbed selenium. Using the 84% absorption reported in [85] and the median Se intake in the SSIS, as much as 284 mg could be distributed to the tissue pool. While there is little information regarding Se distribution at acutely toxic intakes, there can be no doubt that the intakes of 22 to 32 mg per day by victims of the TBF outbreak would have overwhelmed the 15 mg whole-body Se store in a healthy adult; and would have continued to do so for over 100 days post exposure substantially disrupting Se homeostasis for an extended period. Sixty-four percent of the SSIS subjects, in whom we measured their return to baseline, were below the expected normal TNSe concentration. This suggests a long-term, dose-dependent perturbed Se homeostasis (Figure 2B). Furthermore, the emerging evidence that chronic Se toxicity may occur at Se intakes previously considered benign, and potentially increase the risk of chronic disease, calls into question the widespread use of dietary Se supplements in the U.S.

We agree with Ashar’s invited commentary regarding the TBF Se toxicity outbreak [66] in which he concluded: “The time has come for lawmakers to re-evaluate the Dietary Supplement Health and Education Act.” Arguably the necessity for such a re-evaluation is even more imperative in light of a recent report from the Physicians’ Health Study [86] finding that the risk of total cancer was modestly reduced by the daily use of multivitamins/minerals. The authors point out that this finding is contrary to recent recommendations from both the 2010 Dietary Guidelines for Americans [87] and the NIH-sponsored State-of-the-Science Conference [88] that there is no evidence that use of multivitamins reduces the risk of chronic disease. It is not difficult to imagine that the positive multivitamin/mineral finding from the Physicians’ Health Study could motivate new start-up dietary supplement companies marketing their unregulated products via the Internet to a growing number of potential customers. “Let the Buyer Beware” is no longer an adequate national policy to regulate the booming dietary supplement enterprise.
